# Acupuncture for cancer pain: a scoping review of systematic reviews and meta-analyses

**DOI:** 10.3389/fonc.2023.1169458

**Published:** 2023-05-15

**Authors:** Yanji Zhang, Yingrong Zhang, Suzhen Liu, Bocun Li, Zhe Song, Qi Han, Chang Wang, Yiwei Wang, Yanxin Yu, Hongjie Xia, Chun Wang, Jia Li

**Affiliations:** ^1^ College of Acupuncture and Orthopedics, Hubei University of Chinese Medicine, Wuhan, Hubei, China; ^2^ School of Biomedical Engineering and Imaging, Hubei University of Science and Technology, Xianning, Hubei, China; ^3^ Xianning Central Hospital, The First Affiliated Hospital of Hubei University of Science and Technology, Xianning, Hubei, China; ^4^ Faculty of Medicine and Dentistry, University of Alberta, Edmonton, Alberta, AB, Canada

**Keywords:** acupuncture, cancer pain, scoping review, systematic reviews and meta-analyses, alternative medicine (AM)

## Abstract

**Background:**

Due to the effectiveness and safety, acupuncture, one of the traditional therapies of Chinese medicine, has been widely used in clinical practice globally. A few systematic review or meta-analyses have proved its effectiveness and safety towards patients with cancer pain, while there are no syntheses among those evidence. The aim of this scoping review is to summarize the evidence from systematic reviews of acupuncture for the treatment of cancer pain and evaluate the breadth and methodological quality of these evidence as well.

**Methods:**

The scoping review process was guided by the methodology framework of Preferred Reporting Items for Systematic Reviews and Meta-Analyses extension for scoping reviews (PRISMA ScR) and “Arkseyand O’Malley six-stage framework”. Electronic searches were carried out in several online databases from inception to Jan 2022. Systematic reviews and meta-analyses that involve any type of acupuncture for patients with cancer pain will be included. A pair of reviewers independently screened full texts. Moreover, review characteristics were extracted, and methodological quality was assessed using the AMSTAR 2 tool.

**Results:**

Twenty-five systematic reviews and meta-analyses were included. Manual acupuncture is the most frequently included types of test group intervention (48%), followed by acupuncture + medicine (28%), and auricular acupuncture (12%). All the reviews have declared that acupuncture is an effective method for cancer pain treatment. Eleven reviews (44%) aiming at evaluating the safety also have confirmed that acupuncture is safe for treating cancer pain. However, most included studies were conducted in China. With certain geographical limitations, the findings were not representative within the region. The results of our review may owe to the synthesis of all kinds of cancer pain, and only 2 reviews described the type of cancer pain in detail.

**Conclusions:**

This scoping review synthesizes and evaluates existing evidence of acupuncture for cancer pain. From this scoping review of systematic reviews and meta-analyses, there are clear recommendations for future studies: expanding the region of research in the world and trying to conduct the study of different types of cancer pain in details as much as possible. Evidences of acupuncture for cancer pain can inform clinical decision-making.

**Systematic review registration:**

https://inplasy.com/inplasy-2022-1-0073/, identifier INPLASY202210073.

## Introduction

Cancer is the second leading cause of death in the world (1), and about 70% of cancer patients suffer from the great cancer pain, depressing symptom, and so on. Cancer pain, one of the most common and difficult symptoms to control, is the sensation caused by the information that needs to be repaired or regulated by the pain site to the nerve center (2). In addition, cancer pain can interfere with most aspects of the patients, including lives, such as daily activities, cognitive function, sleep quality, and even their emotional and psychological health. Although numerous studies have proved that nearly 50% of cancer patients can control the pain symptom, pain in many patients is still not adequately controlled. The lack of effective pain control can adversely affect the prognosis and life quality of patients (3, 4).

Cancer pain management includes pharmacologic and non-pharmacologic interventions (5). Opioids, anti-convulsants, and nonsteroidal anti-inflammatory drugs (NSAIDs) belong to the pharmacologic methods, and they are used for somatic pain, neuropathic pain, and moderate pain. What’s more, antidepressants, anxiolytics, and steroids are also used to control cancer pain if necessary (6). The World Health Organization (WHO) has highly recommended using opioids in cancer pain management for their advantageous analgesic effect, multiple routes of administration, and ease of titration. Opioids are suitable for advanced cancer and intractable pain patients due to their potent effect (7). However, the adverse effects of analgesic drugs cannot be missed, as they may cause analgesic tolerance development, physical dependence and addiction to drugs, constipation, nausea and vomiting, and respiratory depression (8). The non-pharmacologic interventions mainly include physical therapy, occupational therapy, acupuncture, massage, music therapy, nerve blocks, neuraxial infusion, cognitive behavioral therapy and neurostimulation therapies (5). The non-pharmacologic interventions can not only reduce the pain in cancer patients effectively, but also have positive and long-term effects on their anxiety and fatigue (9).

Acupuncture, a modality coming from Traditional Chinese Medicine (TCM), has been widely used in clinical practice in China. Besides, acupuncture is strongly supported for decreased pain syndrome, such as chronic pelvic pain syndrome, low back pain, chronic scrotal pain, and pain-predominant chronic multi-symptom illness. Furthermore, acupuncture, as part of non-pharmacologic interventions, has been recommended to manage cancer pain by the American Society for Clinical Oncology and the National Comprehensive Cancer Network (10). Recently, an increasing number of clinical studies supported acupuncture’s analgesic effect on cancer pain (11–15). Most importantly, substantial evidence has shown that acupuncture and medication are indistinguishable in cancer pain management and also reduce the adverse effect compared to western medicine (8).

The evidence-based guideline provides a strong recommendation for the treatment of acupuncture to relieve pain in patients with moderate to severe cancer pain (16). Although acupuncture has the record of safety and validity in cancer pain, there still remains a controversial treatment for cancer pain, largely owning to the lack of high-quality systematic evaluation. As a result, this scoping review was conducted to summarize the current evidence on the effectiveness and safety of acupuncture for treating cancer pain and evaluating the quality and bias of the systematic reviews (SRs) and meta-analyses reviewed to identify the future research directions.

## Methods

The following scoping review was performed in accordance with the Preferred Reporting Items for Systematic Reviews and Meta-Analyses extension for scoping reviews (PRISMA ScR) (17) (see **Supplementary Material 1**). The proposed scoping review performed in accordance with the methodology framework of Arksey and O’Malley. This methodology consists of six stages: (1) identifying the research question, (2) identifying relevant studies, (3) studying selection, (4) charting the data, (5) collating, summarizing, and reporting the findings and (6) consulting with key stakeholders (optional). Scooping review protocol is registered in INPLASY (202210073; DOI: 10.37766/inplasy2022.1.0073).

***Step 1: Identifying the research question***

Due to the comprehensive aspect of scoping review, a set of key objectives were identified, as follows:

• To map descriptions (including definitions and characteristics) of the use of acupuncture in treating cancer pain.
• To examine the methodologies and extent to which acupuncture has been applied in treating cancer pain.
• To investigate the impacts of the application of for cancer pain.
• To determine if there are any gaps in researching and identifying the directions for future research.

***Step 2: Identifying relevant studies***

### Search strategy

This review was conducted entirely using electronic databases. The following databases: Cochrane Database, Web of Science, PubMed, Embase, CENTRAL, China National Knowledge Infrastructure (CNKI), China Science and Technology Journal Database (VIP), China Biology Medicine disc (CBMdisc), Wanfang Database, Japan Science and Technology Information Aggregator Electronic, RISS, and KISS were used: and searched from inception to Jan 2022. Title, abstract and keyword fields were searched using a combination of the following terms: “cancer pain”, “acupuncture”, “moxibustion”, and their synonyms. An example of the search strategy for PubMed was included (**Table 1**). The search strategy (see **Supplementary Material 2**) was designed using a broad definition of acupuncture to take the heterogeneity of this concept in research into account. The reference lists of the included articles were also reviewed to ensure that all relevant articles have been included.

**Table 1:** Search strategy for PubMed.

#1 cancer pain [Mesh Terms]
#2 Cancer-Related Pain [Title/Abstract] OR Neoplasm Associated Pain [Title/Abstract] OR Cancer-Associated Pain [Title/Abstract] OR Cancer Related Pains [Title/Abstract] OR Tumor-Associated Pain [Title/Abstract] OR Cancer Associated Pain [Title/Abstract] OR Oncological Pain [Title/Abstract] OR Oncological Pains [Title/Abstract]
#3 #1 OR #2
#4 Acupuncture [Mesh Terms]
#5 Acupuncture Points [Mesh Terms]
#6 Acupuncture, Ear [Mesh Terms]
#7Acupuncture Analgesia [Mesh Terms]
#8Acupuncture Therapy [Mesh Terms]
#9 Auriculotherapy [Mesh Terms]
#10Acupuncture[Title/Abstract] OR acustimulation [Title/Abstract] OR triggerpoint [Title/Abstract] OR Acupuncture Analgesia[Title/Abstract] OR silver needle[Title/Abstract] OR moxibustion[Title/Abstract]OR de qi [Title/Abstract] OR electro-acupuncture[Title/Abstract] OR meridian[Title/Abstract] OR Auriculotherapy [Title/Abstract] OR Extra points[Title/Abstract] OR needle pricking[Title/Abstract]OR Transcutaneous Electric Nerve Stimulation[Title/Abstract] OR acupressure[Title/Abstract] OR needling[Title/Abstract] OR intradermal needle[Title/Abstract] OR Point application[Title/Abstract] OR fire needle[Title/Abstract] OR three-edged needle [Title/Abstract] OR a-shi point[Title/Abstract] OR five phase points[Title/Abstract] OR needle-embedding [Title/Abstract] OR pricking therapy[Title/Abstract] OR point injection [Title/Abstract] OR incision therapy [Title/Abstract]
#11 #4 OR #5 OR #6 OR #7 OR #8 OR #9 OR #10
#12 meta-analysis [Publication Type]
#13 Systematic Review [Publication Type]
#14 systematic reviews as topic [Mesh]
#15 meta-analysis as topic [Mesh]
#16 Systematic review [Title/Abstract] OR meta analysis [Title/Abstract] OR meta-analysis [Title/Abstract] OR meta-analyses [Title/Abstract]
#17 #12 OR #13 OR #14 OR #15 OR #16
#18 #3 AND #11 AND #17

The PICOS framework (population, intervention, comparison, outcomes, and study designs) was followed, as shown in **Table 2**. Studies was included if they met the following criteria: (1) Systematic reviews and Meta-analysis; (2) Preventions were focused on examining acupuncture or moxibustion for the treatment of cancer pain. The definition of acupuncture and intervention types is displayed in **Table 3**; (3) Primary outcomes included pain relief rate, VAS scores, Pain Numerical Rating Scale (NRS) score, Analgesic efficacy, EORTC QLQ-C30 scores, etc. Studies were excluded if: (1) Systematic reviews do not included RCTs (randomized control trial) or qRCTs (quasi-randomized control trial). (2) Publications were not full reports; (3) Protocol of reviews.

**Table 2:** PICOS framework.

Population	Patients diagnosed with cancer pain.
Intervention	Treatment group intervention at least includes a kind of acupuncture therapy (acupuncture, electroacupuncture, auricular acupuncture, etc.) or moxibustion therapy.
Comparison	Any comparators.
Outcome	Effectiveness indicators: total effective rate; pain relief rate, VAS scores, Pain Numerical Rating Scale (NRS) score, Analgesic efficacy, EORTC QLQ-C30 scores, etc.
Study design	Systematic review or meta-analyses that only included RCTs or qRCTs.

**Table 3:** Definitions of modalities of acupuncture and related therapies in this overview of systematic review.

Type of intervention	Definition
Manual acupuncture	A traditional Chinese method for treating illness, which uses special thin needles to push into the skin in particular parts of the body.
Auricular acupuncture	One kind of acupuncture treatment method, and place fine needles in specifically designated “puncture points” on the external ear.
Acupoint injection	A method of treating disease by injecting certain medical liquids of TCM drugs or western drugs into certain acupoints.
Electroacupuncture	One type of modern acupuncture technique, and the needle is attached to a trace pulse current after it is inserted into the selected acupoint to produce the synthetic effect of electric and needling stimulation.
Transcutaneous electric nerve stimulation (TENS)	TENS is a non-invasive method, involving the use of a mild electrical current produced by a device to stimulate the nerves in acupoints for treating effect.
Wrist-ankle acupuncture	A modern subcutaneous acupuncture technique that is superficial acupuncture and applied on the specific area of the wrist or the ankle corresponding to the site of pain area to treat a range of pain symptoms throughout the body.
Acupressure	Acupoint pressing is a TCM therapy used with the pulp of the index finger and middle finger to press the specific acupoints, which has the effect of freeing the channels and networks vessels.
Fire needle	Fire needle is a special acupuncture therapy method that uses a needle made of a special material to burn red on the fire, and then quickly stabs the specific parts of the body and acupoints to achieve the purpose of curing diseases.

***Step 3: Study selection***

### Inclusion criteria

Studies that meet the following criteria were included:

(1) Type of studies. Systematic reviews and meta-analyses that examined the effectiveness or safety of acupuncture and related therapies for treating cancer pain.
(2) Type of participants. We included patients diagnosed with cancer pain.
(3) Type of interventions. The treatment group intervention of clinical research at least included a kind of acupuncture therapy (manual acupuncture, electroacupuncture, auricular acupuncture, etc.) or moxibustion therapy.
(4) Type of comparators. There is no limitation about the type of comparators.
(5) Types of outcome measures. Primary outcomes included pain relief rate, VAS scores, Pain Numerical Rating Scale (NRS) score, Analgesic efficacy, EORTC QLQ-C30 scores, etc.

### Exclusion criteria

Studies were excluded if:

(1) Duplicated. The research team eliminated the duplicates, due to the possibility of the paper appearing more than one time;
(2) Publications were not full reports;
(3) Protocol of research;

A two-step study selection was used in this review. A first exclusion by title and abstract was made using the inclusion/exclusion criteria by author A (Yanji Zhang) and B (Yingrong Zhang). Subsequently, 2 other researchers (Suzhen Liu and Yiwei Wang) retrieved the full-text for further screening to determine whether the studies should be included or excluded. If there is a disagreement, we would have a discussion with another people (Jia Li) in our panel.

***Step 4: Charting the data***

A standardized set of data extraction items guided by our research question were developed and piloted at the protocol stage to extract key data from the included studies. Data extraction was performed by author A (Yanxin Yu) and B (Hongjie Xia), and checked by anther author (Chang Wang and Chun Wang).

Variables were extracted for the following key groupings: 1. identifying information: Title, article language, the author of article, institution, publication date, nationality, publication journal, and type of funding; 2. Characteristics of intended participants: patient’s age, patient’s gender, and sample size; 3. Methodological details: study design, type of interventions, type of comparators, and types of outcome measures; 4. Results and discussion: significant findings, conclusions, limitations, and suggestion for future research. A preliminary set of data extraction items for this protocol are shown in **Table 4**.

**Table 4:** Preliminary standardized data extraction items.

1.Publication details	Study title List of authors
	Article language
	Institution
	Year of publication
	Country
	Publisher
	Type of funding
2.Characteristics of intended participants	Age
	Sex
	Sample size
3.Methodological details	Study design
	Type of interventions
	Type of comparators
	Types of outcome measures
4.Results and discussion	Significant findings
	Conclusions
	Limitations
	Suggestion for future research

***Step 5: Collating, summarizing, and reporting the findings***

Analysis: Both quantitative analysis based on numerical counts (i.e., characteristics of included studies, analysis of the main outcome) and qualitative analysis through a narrative synthesis were provided. The quantitative analysis used the data from papers, focusing on the characteristics of the use of acupuncture in the treatment of cancer pain, the methodological details of clinical research of acupuncture for cancer pain, and the impact of the application of acupuncture for cancer pain. For the qualitative analysis, the focus is on the discussion of the studies as a way to determine if there are any gaps in researching and identifying the directions for future research.

Reporting: The data extracted from the study are presented in the form of tables and pictures. The results were briefly organized into a tabular format and analyzed using a narrative description. As scoping review research, there is no plan for subgroup analysis and sensitivity analysis of data.

***Step 6: Expert consulting***

Though this step is an optional one, it was applied to identify the gaps in our literature review. To enhance this scoping review, we relied on the expertise and experience of acupuncturists and methodological experts by providing feedback on it. Suggestions were taken into consideration to contribute to this scoping review.

***Additional Step: Quality assessment:***

Two researchers (Bocun Li and Yiwei Wang) evaluated the quality of included studies by using the AMSTAR2 tool in duplicate. Any disagreement was resolved by a third investigator (Yanxin Yu). The AMSTAR2 scale contains a total of 16 entries: Each entry is answered as “yes” or “no”, and some entries can be answered as “partial yes”. If no items are defective or there is only one non-key item that is defective, the methodological quality of the commented SR is high. When more than one non-key item is defective and no key item is defective, the methodological quality is judged into medium. When a key item is defective with or without non-critical item defects, the methodological quality is low. The methodological quality is extremely low when there is more than one key item defect, with or without non-critical item defects.

## Results

### Summary of included studies

**Figure 1:**
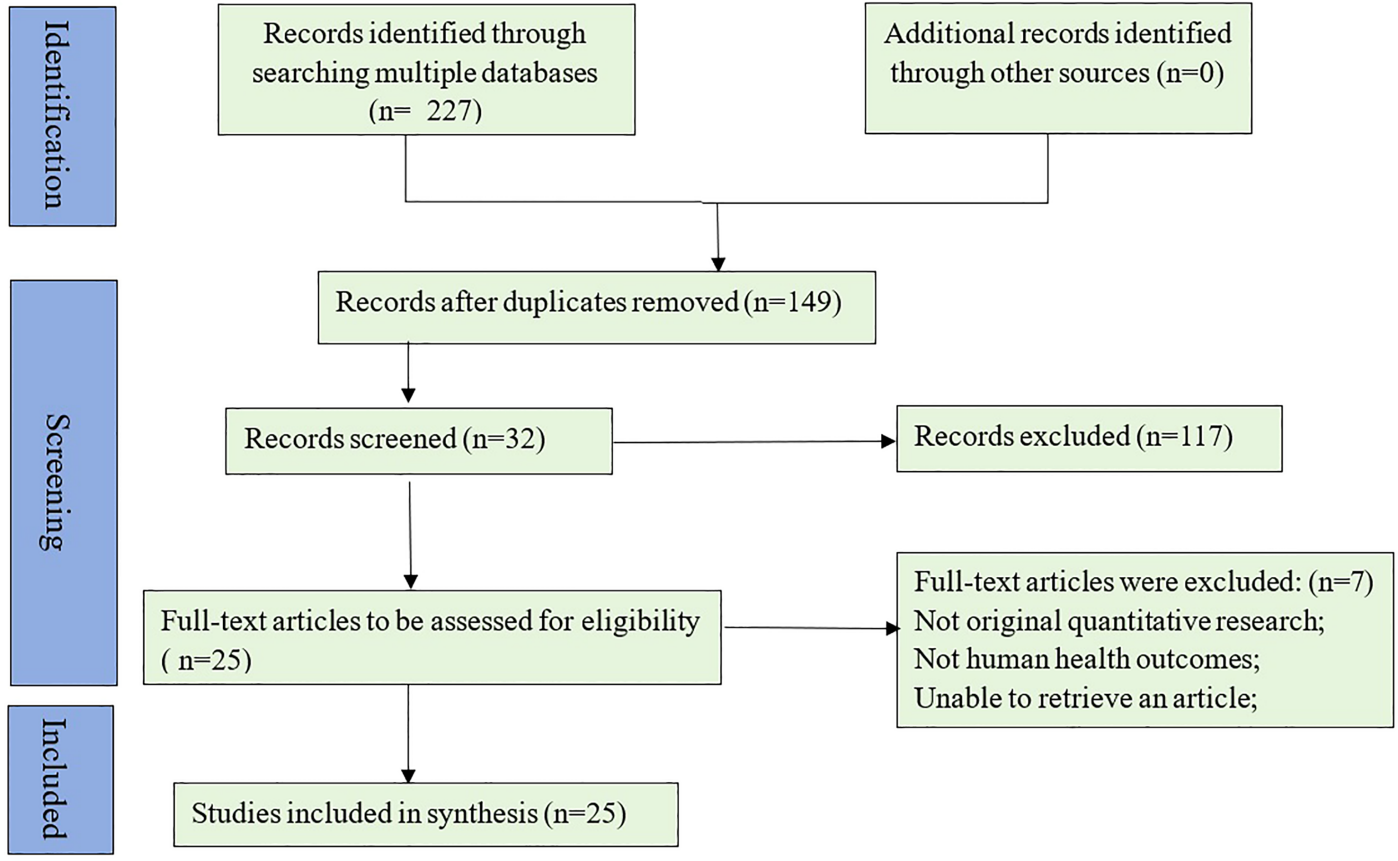
PRISMA 2020 flow diagram study selection.

A total of 227 articles were found (28 in PubMed, 4 in Cochrane Database, 2 in Web of Science, 109 in Embase, 40 in CNKI, 7 in VIP, 17 in CBMdisc, and 20 in Wanfang Database). Duplicates were excluded and 149 citations were exported to EndNote (EndNote X9, Thomson Reuters, New York, USA). The first exclusion by title and abstract was made by using the inclusion/exclusion criteria, by author Yanxin Yu and author Hongjie Xia. Afterwards, the 2 same researchers retrieved the full text for further screening to determine whether studies should be included or excluded. If there is a disagreement, we would have a discussion with other people (Jia Li) in our panel. Totally, 32 articles were reserved following inclusion criteria and found the full-text. Meanwhile, 1 article was the animal model experiment (18), 2 articles were the letter’s response to the author (19, 20), and 3 protocols were verified (21–23), so there were 25 articles for the scoping reviews (see **Figure 1**). Only 2 included articles did not conduct a meta-analysis, while 23 reviews have done the meta-analysis to determine the outcomes. The median number of the used databases was 7.5, and seven databases were commonly used (12/25), with a range of 5-14 databases. Most reviews were conducted from the inception of databases, and 5 reviews from the specific date of databases, such as 1950, 1966, 1986, 1999, and 2005 (24–28).

### Population, interventions, comparators, and outcomes of included reviews

**Figure 2:**
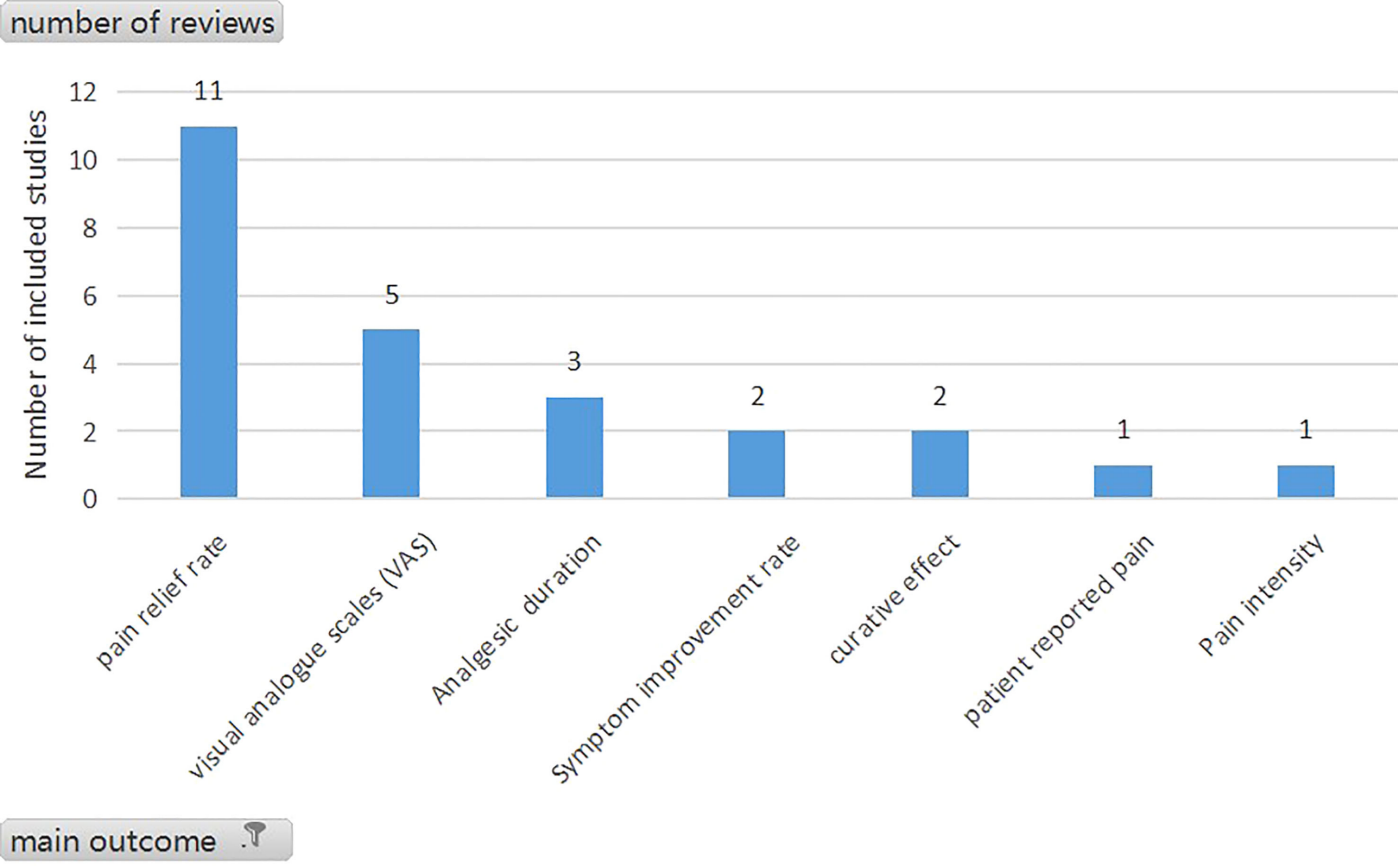
Analyze of main outcome(n=25).

The summary of population, interventions, comparators, and outcomes of included reviews are shown in **Table 5**. **Supplementary material 3** presents the characteristics of each review, and **Figure 2** displays the assessed outcome. All the articles had no restriction on gender. **Table 4** shows that 56.00% of reviews had no restriction on age (14/25), and 44.00% of reviews paid more attention to adults (11/25).

**Table 5:** Summary of characteristics of included studies.

1.Population	n	%
adult	11	44.00%
no restriction	14	56.00%
2.Intervention		
Test group		
acupuncture	12	48.00%
acupuncture + medicine	7	28.00%
auricular acupuncture	3	11.54%
Transcutaneous electric nerve stimulation (TENS)	2	8.00%
acupoint injection	2	8.00%
wrist-ankle acupuncture + medicine	2	8.00%
wrist-ankle acupuncture	2	8.00%
electroacupuncture	2	8.00%
auricular acupuncture + medicine	2	8.00%
catgut embedding at acupoint + medicine	2	8.00%
wrist-ankle acupuncture + medicine	1	4.00%
fire needle + medicine	1	4.00%
electroacupuncture+ medicine	1	4.00%
auricular injection + medicine	1	4.00%
auricular injection	1	4.00%
auricular acupuncture + medicine	1	4.00%
acupuncture + acupoint injection	1	4.00%
acupressure	1	4.00%
acupoint injection + medicine	1	4.00%
Control group		
Placebo acupuncture	12	48.00%
medicine	12	48.00%
analgesics	5	20.00%
three-step analgesia medicine	3	12.00%
sham acupuncture	2	8.00%
Blank control	2	8.00%
sham electroacupuncture	1	4.00%
Radiotherapy	1	4.00%
non-drug injection	1	4.00%
drug intramuscular injection	1	4.00%
chemotherapy	1	4.00%
3.Study designs		
only RCT	18	69.23%
RCT and qRCT	7	26.92%
4.Component assessed within reviews		
evaluation the efficacy	25	100.00%
evaluation the safety	10	40.00%

All the interventions are presented in **Table 4**: Manual acupuncture is the most frequently included types of test group intervention (n=12), about 50%, followed by acupuncture + medicine (n=7), auricular acupuncture (n=3), acupoint injection, transcutaneous electric nerve stimulation (TENS), wrist-ankle acupuncture + medicine, wrist-ankle acupuncture, electroacupuncture, auricular acupuncture + medicine, and catgut embedding at acupoint + medicine (n=2). Other interventions only refer to one time. Meanwhile, different types of acupuncture (e.g. fire needle) and different kinds of auricular acupuncture (needle, pill, and injection) were mentioned. A total of 12 reviews mentioned medicine as the control group (15, 24, 26, 27, 29–37), 12 reviews described placebo acupuncture as the control group (15, 24, 31–33, 36, 38–43) and 5 reviews clearly showed the analgesics as the control group (28, 31, 38, 42, 44). Three studies included three-step analgesia medicine (25, 45, 46), blank control appeared in 2 articles (43, 47), sham acupuncture was in 2 reviews (32, 38), and 1 review included sham electroacupuncture (31), radiotherapy and chemotherapy appeared (42). The duration of the session ranged from 7 days to 6 weeks. Most timing of assessment is 7 days or 14 days. All the included RCTs/qRCTs in reviews didn’t set up a follow-up period to observe long-term efficacy.

The included reviews comprised the following study designs: RCT(18/25) (24–26, 28, 29, 31, 33, 36–45, 47), RCT and qRCT (7/25) (15, 27, 30, 32, 34, 35, 46). 22 reviews did not show the specific cancer pain diagnosis standard, and only 3 reviews presented disease diagnosis standard (25, 36, 41). As the type of cancer, 23 reviews showed multi-type cancer, and only 2 reviews referred to specific cancer, lung cancer and liver cancer (28, 46).

Totally, all reviews assessed efficacy, and only about 40.00% (10/25) reported safety (15, 25–29, 36, 39, 42, 47). Many reviews required included studies to contain either a efficacy or safety-related outcome (10/25) (15, 25–29, 36, 39, 42, 47).

**Figure 3:**
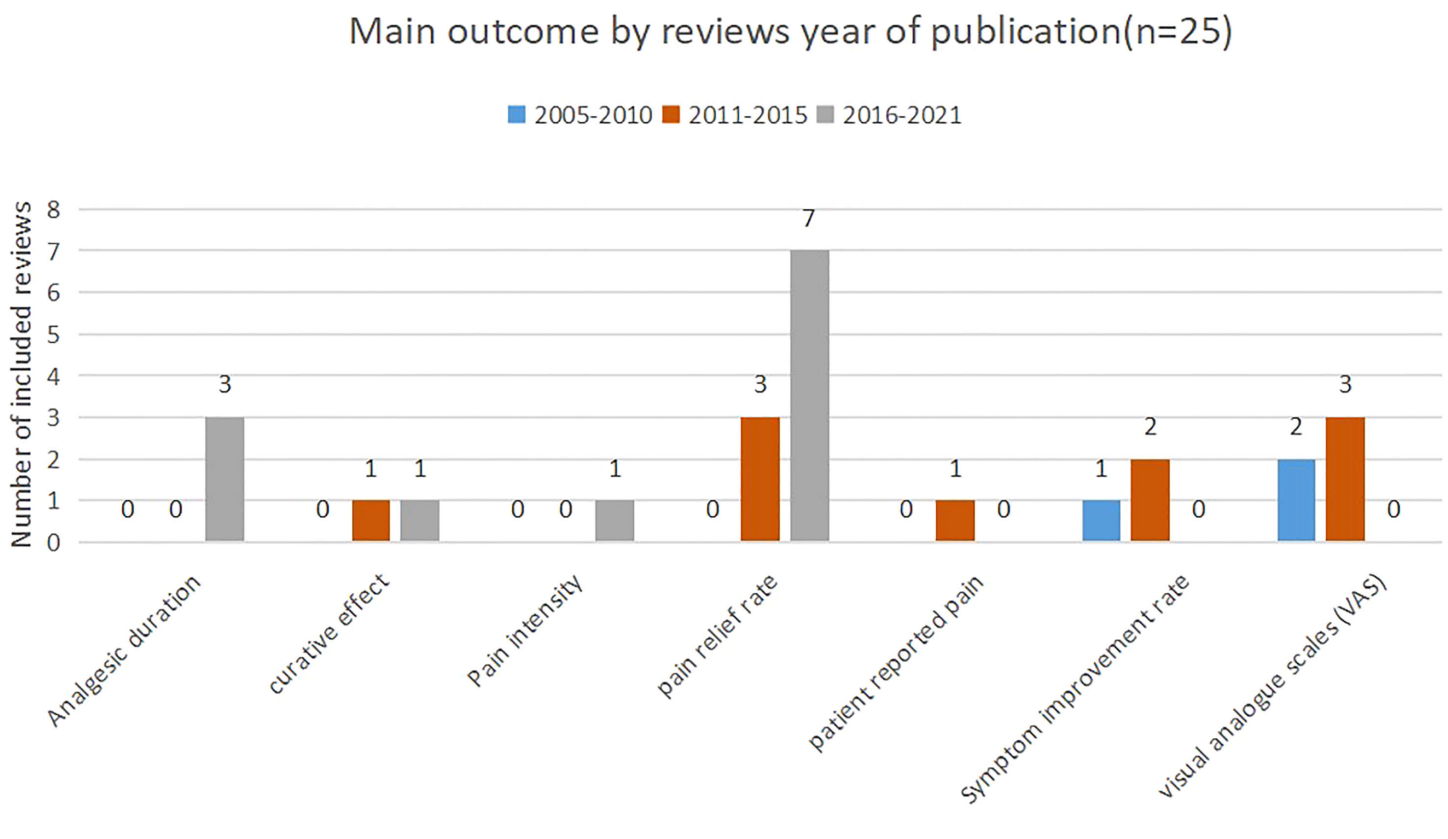
Main outcome by reviews year of publication (n=25).

As shown in **Figure 2**, pain relief rate was the most frequently assessed outcome in reviews (n=11), closely followed by visual analogue scales (VAS) (5/25) and analgesia duration (3/25). **Figure 3** shows that pain relief rate was not the main outcome between 2005 and 2010. After 2011, pain relief rate has become the commonly used main outcome, and after 2016 pain relief rate was the most frequently assessed outcome in reviews (n=7).

### Quality assessment

The quality of the systematic reviews and meta-analyses as determined by AMSTAR 2 is presented in **Table 6**. All reviews have done search strategy comprehensively, and all the reviews have been screened by 2 reviewers independently. However, 9 reviews didn’t clarify the reason for excluding articles and 1 review did not explain the information in details (38). Two reviews didn’t carry out the meta-analysis and did not have adequate information to analyze the risk of bias of included clinical studies (30, 47). Only 1 review indicated that their review was registered with PROSPERO (38), 22 reviews used a tool that addressed all the risk of bias components, and 4 reviews did not (28, 30, 35, 47). However, nearly 95% of included RCTs/qRCTs in review didn’t report blinding of participants/investigators and had a high risk.

**Table 6:** Quality of included reviews based on AMSTAR (n=25).

First Author	PICO	review methods	study selection	study stratage	Date Extraction	Excluded studies	Describe Studies	ROB Tools	Report Funding	Statistical Methods	ROB assessment	ROB Discussion	Study Differences	Heterogeneity assessment	Reporting Biases	COI and Funding
De-hui Li (29)	Yes	No	No	Yes	Yes	No	Yes	Yes	No	Yes	Yes	Yes	Yes	No	No	No
Bei Dong (44)	Yes	No	No	Yes	Yes	No	Yes	Yes	No	Yes	Yes	Yes	Yes	Yes	Yes	No
Yihan He (38)	Yes	Yes	No	Yes	Yes	No	No	Yes	No	Yes	Yes	Yes	Yes	Yes	Yes	No
Juan Yang (30)	Yes	No	No	Yes	Yes	No	Yes	No	No	No meta-analysis conducted	No meta-analysis conducted	No meta-analysis conducted	No meta-analysis conducted	No meta-analysis conducted	No meta-analysis conducted	No
Caiqiong Hu (39)	Yes	No	No	Yes	Yes	No	Yes	Yes	No	Yes	Yes	Yes	Yes	Yes	Yes	No
Yulan Yang (15)	Yes	No	Yes	Yes	Yes	Yes	Yes	Yes	Yes	Yes	Yes	Yes	Yes	No	No	Yes
Carole A Paley (31)	Yes	No	Yes	Yes	Yes	Yes	Yes	Yes	No	Yes	Yes	Yes	Yes	Yes	Yes	Yes
Adam Hurlow (24)	Yes	No	Yes	Yes	Yes	Yes	Yes	Yes	No	No meta-analysis conducted	No meta-analysis conducted	No meta-analysis conducted	No meta-analysis conducted	No meta-analysis conducted	No meta-analysis conducted	Yes
Tae-Young Choi (32)	Yes	No	Yes	Yes	Yes	Yes	Yes	Yes	Yes	No meta-analysis conducted	No meta-analysis conducted	No meta-analysis conducted	No meta-analysis conducted	No meta-analysis conducted	No meta-analysis conducted	Yes
Carole A Paley (33)	Yes	No	Yes	Yes	Yes	Yes	Yes	Yes	No	Yes	No	No	Yes	Yes	No	Yes
Carole A Paley (40)	Yes	No	Yes	Yes	Yes	Yes	Yes	Yes	No	No meta-analysis conducted	No meta-analysis conducted	No meta-analysis conducted	No meta-analysis conducted	No meta-analysis conducted	No meta-analysis conducted	Yes
Karen A Robb (41)	Yes	No	Yes	Yes	Yes	Yes	Yes	Yes	No	No meta-analysis conducted	No meta-analysis conducted	No meta-analysis conducted	No meta-analysis conducted	No meta-analysis conducted	No meta-analysis conducted	Yes
Hyangsook Lee (42)	Yes	No	Yes	Yes	Yes	Yes	Yes	Yes	No	Yes	Yes	Yes	No	Yes	No	Yes
Pu Yang Lee (45)	Yes	No	No	Yes	Yes	No	Yes	Yes	No	Yes	Yes	Yes	Yes	Yes	No	No
Hao Peng (47)	Yes	No	No	Yes	Yes	No	Yes	No	No	No meta-analysis conducted	No meta-analysis conducted	No meta-analysis conducted	No meta-analysis conducted	No meta-analysis conducted	No meta-analysis conducted	No
Jianfeng Wang (25)	Yes	No	No	Yes	Yes	No	Yes	Yes	No	Yes	Yes	Yes	Yes	No	No	No
Jie Zhou (26)	Yes	No	No	Yes	Yes	No	Yes	Yes	No	Yes	No	No	No	No	No	No
Zou Yu (34)	Yes	No	Yes	Yes	Yes	Yes	Yes	Yes	No	Yes	Yes	Yes	Yes	No	Yes	No
Sun Ge (35)	Yes	No	Yes	Yes	Yes	Yes	Yes	No	No	Yes	No	No	No	Yes	No	No
Zheng Yi (27)	Yes	No	Yes	Yes	Yes	Yes	Yes	Yes	No	Yes	Yes	Yes	Yes	No	Yes	No
Zhou Jie (36)	Yes	No	Yes	Yes	Yes	Yes	Yes	Yes	Yes	Yes	No	No	No	No	No	Yes
HU Cai-qiong (43)	Yes	No	Yes	Yes	Yes	Yes	Yes	Yes	No	Yes	Yes	Yes	Yes	No	No	No
SUN Qi-zhe (28)	Yes	No	Yes	Yes	Yes	Yes	Yes	No	No	Yes	No	No	No	No	No	No
BIAN Shuang-lin (46)	Yes	No	Yes	Yes	Yes	Yes	Yes	Yes	No	Yes	Yes	Yes	Yes	No	No	No
CHEN Ting-yu (37)	Yes	No	Yes	Yes	Yes	Yes	Yes	Yes	No	Yes	Yes	Yes	Yes	No	No	No

### Systematic review findings

All the reviews have declared that acupuncture is an effective method for cancer pain treatment. Totally, 11 reviews aiming at evaluating the safety also have confirmed that acupuncture is safe for treating cancer pain. On the other hand, compared with western medicine, acupuncture has a greater advantage in security and can reduce side effects happening, such as nausea, vomiting, constipation, and dizziness. Nearly all the included interventions can mitigate the degree of pain, such as manual acupuncture, sham acupuncture, wrist-ankle acupuncture, conventional care, and so on. **Table 7** shows the findings from included studies.

**Table 7:** Findings of included studies.

Group	Outcome	Finding
Acupuncture VS Medicine	the analgesic effect validated with a pain measurement	MD, RR 0.95, 95%CI (0.85 to 1.07)
	Effectiveness of analgesic efficacy	SMD, RR 1.11, 95%CI (0.97 to 1.26)
	pain reduction	SMD, RR 1.12, 95%CI (0.98 to 1.28)
Wrist-ankle acupuncture+Conventional care group VS Conventional care group	VAS	SMD or WMD, RR 1.12, 95%CI (0.92 to 1.36)

RR, relative risk; MD, Standard Mean Difference; WMD, Weighted Mean Difference; SMD, Standard Mean Difference; CI, confidence interval.

### Recommendations for future reviews

The 18 reviews included studies primarily conducted in China, and researchers in the UK conducted 5 reviews. Most reviews did not describe the diagnosis of cancer pain, and only 3 articles have done; 23 studies included multi-type cancer, and 2 articles refer to liver cancer and lung cancer. As for the adverse events, only 6 reviews related, and 2 reviews explained the adverse reactions and numbers of patients in details. (**Table 8**)

**Table 8:** Methodological issues present in included reviews.

Methodological	No. of reviews	References
Included studies primarily conducted in China	17	Previous studies (15, 25–29, 34–39, 43–47),
Included studies clearly describe diagnosis of cancer pain	3	Previous studies (25, 36, 41)
Included studies clearly describe the type of cancer	2	Previous studies (28, 46)
Included studies clearly describe the adverse events and the number of patients	2	Previous studies (25, 29)

### Consulting results from experts

Six experts were consulted and provided advice. All experts agreed that acupuncture is an effective and safety method for cancer pain treatment. More than two-thirds of experts believed that electroacupuncture has a better significant effect on cancer pain among these acupuncture therapies. Two experts mentioned that attention should be paid to the combined application of acupuncture therapies in the clinical practice of acupuncture for cancer pain.

## Discussion

In this scoping review, 25 systematic reviews of acupuncture for cancer pain management were included, which evaluated numerous primary studies with various study designs and qualities. They were published from 2005 to 2021: 18 were conducted in China, 5 in the UK (24, 31, 33, 40, 41), 2 in Korea (32, 42), and 1 in the USA (30). And, 24 out of 26 included reviews that covered multi-types of cancer; 1 included liver cancer from 2005 to 2021 (28), and 1 systematic review of lung-cancer-only reported negative results, without positive results from others (31). The methodological quality of included evidence was relatively poor. Only 2 systematic reviews (15, 39) we included were deemed of moderate or high methodological quality and 23 systematic reviews were deemed to be quietly low. Overall, the synthesis of these systematic reviews was broad, and there are lots of opportunities to improve the qualities of future evidence in this field.

### Effect of Acupuncture for cancer pain based on evidence

In terms of therapeutic effect, based on the results of our review, it is confirmed that acupuncture has an accurate effect to alleviate cancer pain. Most original trials indicate the safety and effectiveness of acupuncture targeted to patients suffering from cancer pain. Though the result is encouraging, and the future application of acupuncture is promising, the heterogeneity and the research quality of original studies still seem worrisome.

As for interventions, a few systematic reviews sought a broad definition of acupuncture, and many sorts of acupuncture interventions were included: 20 reviews targeted at intervention compounded of two or more sorts of acupuncture, and only 6 systematic reviews confined to single interventions (24, 30, 31, 39–41). Some reviews reported that compounded intervention of acupuncture has superior effectiveness than a single intervention (32, 44, 48). Comparing different acupuncture types, the number of studies related to auricular acupuncture (n=3) was more than that related to electro-acupuncture (n=2). More reviews focused on the combination of acupuncture therapies and medicine (n=19) than the combination of acupuncture therapies (n=1). As for types of cancer pain, most studies (n=23) included multi-type cancer, only 2 articles refer to liver cancer and lung cancer. Therefore, the evidence of acupuncture in the treatment of cancer pain related to liver cancer and lung cancer is more sufficient. Follow-up clinical studies on acupuncture for cancer pain caused by breast cancer, colorectal cancer and other cancers should be carried out. The therapeutic effects of acupuncture on cancer pain caused by different types of cancer should be compared. At the same time, no studies reported the relative priority levels among manual acupuncture, electro-acupuncture, and auricular acupuncture. No study reported whether there is a synergism or additive effect between diverse acupuncture interventions in cancer pain.

### Strengths and limitations

The strengths of this scoping review are as follows. Methodology based on an accepted framework was used for scoping reviews, with more than 3 researchers assessing the quality and results of included articles in duplicate. Also, a rigorous assessment has done the included reviews using AMSTAR 2. In addition, none of the primary study places restrictions on the age or gender of patients, which increases the representative of the sample enrolled to original SR.

The limitation of this scoping review is that most included studies were conducted in China, especially in Guangdong and Zhejiang Province. With certain geographical limitations, the findings were not representative within the region. More studies need to conducted in other settings by other investigators to determine the true effects of acupuncture. Evidence has proved that different types of cancer pain may have different pain features (49). The results of our review may owe to the synthesis of all kinds of cancer pain, and only 2 reviews described the type of cancer pain in detail. It remains to figure out whether acupuncture has an affinity for certain types of pain. All reviews assessed intervention efficacy, but fewer than a quarter assessed a component, including a reach, engagement, safety, or cost-effectiveness.

## Summary and conclusion

This scoping review synthesizes and evaluates existing evidence of acupuncture for cancer pain. From this scoping review of systematic reviews and meta-analyses, there are clear recommendations for future studies: expanding the region of research in the world and trying to conduct the study of different types of cancer pain in details as much as possible. Evidences of acupuncture for cancer pain can inform clinical decision-making.

## Data availability statement

The original contributions presented in the study are included in the article/**Supplementary Material**. Further inquiries can be directed to the corresponding authors.

## Author contributions

YaZ, YiZ, SL, BL and ZS have contributed equally to this work. JL and ChuW had full access tothe study data and take responsibility for the integrity and accuracy of data analysis. Concept and design: All authors. Acquisition, analysis, or interpretation of data: YaZ, YiZ, JL, SZ, ChuW. Drafting of the manuscript: YaZ, YiZ, SL. Critical revision of the manuscript: JL, ChuW, ChaW. Intellectual content: YaZ, YiZ, SL, BL, ZS, ChW, JL. Statistical analysis: QH, SSL, YiZ, YW, YY, HX. Quality assessment: BL, YW, YY. All authors contributed to the article and approved the submitted version.
