# Supplementary material for: Acupuncture for cancer pain: a scoping review of systematic reviews and meta-analyses
Source: Front Oncol. 2023 May 15;13:1169458. doi: 10.3389/fonc.2023.1169458 (PMC10226720; doi:10.3389/fonc.2023.1169458)
Supplement: Supplementary file 2 [file DataSheet_2.docx]

**Supplementary material 2 search strategy**

**1.PubMed**

#1 cancer pain [Mesh Terms]

#2 Cancer-Related Pain [Title/Abstract] OR Neoplasm Associated Pain [Title/Abstract] OR Cancer-Associated Pain [Title/Abstract] OR Cancer Related Pains [Title/Abstract] OR Tumor-Associated Pain [Title/Abstract] OR Cancer Associated Pain [Title/Abstract] OR Oncological Pain [Title/Abstract] OR Oncological Pains [Title/Abstract]

#3 #1 OR #2

#4 Acupuncture [Mesh Terms]

#5 Acupuncture Points [Mesh Terms]

#6 Acupuncture, Ear [Mesh Terms]

#7Acupuncture Analgesia [Mesh Terms]

#8Acupuncture Therapy [Mesh Terms]

#9 Auriculotherapy [Mesh Terms]

#10Acupuncture[Title/Abstract] OR acustimulation [Title/Abstract] OR triggerpoint [Title/Abstract] OR Acupuncture Analgesia[Title/Abstract] OR silver needle[Title/Abstract] OR moxibustion[Title/Abstract]OR de qi [Title/Abstract] OR electro-acupuncture[Title/Abstract] OR meridian[Title/Abstract] OR Auriculotherapy [Title/Abstract] OR Extra points[Title/Abstract] OR needle pricking[Title/Abstract]OR Transcutaneous Electric Nerve Stimulation[Title/Abstract] OR acupressure[Title/Abstract] OR needling[Title/Abstract] OR intradermal needle[Title/Abstract] OR Point application[Title/Abstract] OR fire needle[Title/Abstract] OR three-edged needle [Title/Abstract] OR a-shi point[Title/Abstract] OR five phase points[Title/Abstract] OR needle-embedding [Title/Abstract] OR pricking therapy[Title/Abstract] OR point injection [Title/Abstract] OR incision therapy [Title/Abstract]

#11 #4 OR #5 OR #6 OR #7 OR #8 OR #9 OR #10

#12 meta-analysis [Publication Type]

#13 Systematic Review [Publication Type]

#14 systematic reviews as topic [Mesh]

#15 meta-analysis as topic [Mesh]

#16 Systematic review [Title/Abstract] OR meta analysis [Title/Abstract] OR meta-analysis [Title/Abstract] OR meta-analyses [Title/Abstract]

#17 #12 OR #13 OR #14 OR #15 OR #16

#18 #3 AND #11 AND #17

**2.EMBASE**

#1 'cancer pain '/exp

#2 'cancer pains '/exp

#3 'Cancer-Related Pain':ti,ab OR 'Neoplasm Associated Pain':ti,ab OR '[Bell palsy](https://www.cochranelibrary.com/advanced-search/mesh#0)':ti,ab OR 'Cancer-Associated Pain':ti,ab OR 'Cancer Related Pains':ti,ab OR 'Tumor-Associated Pain':ti,ab OR 'Cancer Associated Pain':ti,ab OR 'Oncological Pain':ti,ab OR 'Oncological Pains':ti,ab

#4 #1 OR #2 OR #3

#5 'acupuncture'/exp

#6 'acupuncture needle'/exp

#7 'electroacupuncture '/exp

#8 'acupuncture':ti,ab OR 'acustimulation':ti,ab OR 'trigger point':ti,ab OR 'Acupuncture Analgesia':ti,ab OR 'silver needle':ti,ab OR 'moxibustion':ti,ab OR 'de qi':ti,abOR 'electro-acupuncture':ti,ab OR 'meridian':ti,ab OR 'incision therapy':ti,ab OR 'Auriculotherapy':ti,ab OR 'Extra points':ti,ab OR 'needle pricking':ti,ab OR 'Transcutaneous Electric Nerve Stimulation':ti,ab OR 'acupressure':ti,ab OR 'needling':ti,ab OR 'intradermal needle':ti,ab OR 'Point application':ti,ab OR 'fire needle':ti,ab OR 'three-edged needle':ti,ab OR 'a-shipoint':ti,ab OR 'five phase points':ti,ab OR 'needle-embedding':ti,ab OR 'pricking therapy':ti,ab OR 'point injection':ti,ab

#9 #5 OR #6 OR #7 OR #8

#10 'meta-analysis'/exp

#11 'meta-analysis (topic)'/exp

#12 'systematic review'/exp

#13 'systematic review (topic)'/exp

#14 ‘Systematic review’:ti,ab OR 'meta analysis':ti,ab OR 'meta-analysis':ti,ab OR 'metaanalysis':ti,ab OR 'meta-analyses':ti,ab

#15 #10 OR #11 OR #12 OR #13 OR #14

#16 #4 AND #9 AND #15

**3.Cochrane Library**

#1 MeSH descriptor: [cancer pain] explode all trees

#2 'cancer pains':ti,ab OR 'Neoplasm Associated Pain':ti,ab OR 'Cancer-Associated Pain':ti,ab OR 'Cancer Related Pains':ti,ab OR 'Tumor-Associated Pain':ti,ab OR 'Cancer Associated Pain':ti,ab OR 'Oncological Pain':ti,ab OR 'Oncological Pains':ti,ab

#3 #1 OR #2

#4 MeSH descriptor: [Acupuncture] explode all trees

#5 MeSH descriptor: [Acupuncture Therapy] explode all trees

#6 MeSH descriptor: [Acupuncture, Ear] explode all trees

#7 'acupuncture':ti,ab OR 'acustimulation':ti,ab OR 'trigger point':ti,ab OR 'Acupuncture Analgesia':ti,ab OR 'silver needle':ti,ab OR 'moxibustion':ti,ab OR 'de qi':ti,abOR 'electro-acupuncture':ti,ab OR 'meridian':ti,ab OR 'incision therapy':ti,ab OR 'Auriculotherapy':ti,ab OR 'Extra points':ti,ab OR 'needle pricking':ti,ab OR 'Transcutaneous Electric Nerve Stimulation':ti,ab OR 'acupressure':ti,ab OR 'needling':ti,ab OR 'intradermal needle':ti,ab OR 'Point application':ti,ab OR 'fire needle':ti,ab OR 'three-edged needle':ti,ab OR 'a-shipoint':ti,ab OR 'five phase points':ti,ab OR 'needle-embedding':ti,ab OR 'pricking therapy':ti,ab OR 'point injection':ti,ab

#8 #4 OR #5 OR #6 OR #7

#9 MeSH descriptor: [systematic review] explode all trees

#10 MeSH descriptor: [systematic review as Topic] explode all trees

#11 MeSH descriptor: [meta-analysis] explode all trees

#12 MeSH descriptor: [meta-analysis as Topic] explode all trees

#13 #9 OR #10 OR #11 OR #12

#14 #3 AND #8 AND #13

**4.Web of science**

#1TS= (" Cancer pain "[Mesh] OR Cancer-Related Pain OR Neoplasm Associated Pain OR Cancer-Associated Pain OR Cancer Related Pains OR Tumor-Associated Pain OR Cancer Associated Pain OR Oncological Pain OR Oncological Pains )

#2TS=("Acupuncture"[Mesh] OR "Acupuncture Points"[Mesh] OR "Acupuncture, Ear"[Mesh] OR "Acupuncture Analgesia"[Mesh] OR "Acupuncture Therapy"[Mesh] OR "Auriculotherapy"[Mesh] OR Acupuncture OR acustimulation OR triggerpoint OR Acupuncture Analgesia OR silver needle OR moxibustion OR de qi OR electro-acupuncture OR meridian OR Auriculotherapy OR Extra points OR needle pricking OR Transcutaneous Electric Nerve Stimulation OR acupressure OR needling OR intradermal needle OR Point application OR fire needle OR three-edged needle OR a-shi point OR five phase points OR needle-embedding OR pricking therapy OR point injection OR incision therapy)

#3TS= ("meta-analysis" [Publication Type] OR "Systematic Review" [Publication Type] OR "systematic reviews as topic"[Mesh] OR "meta-analysis as topic"[Mesh] OR Systematic review [Title/Abstract] OR meta- analysis [Title/Abstract] OR meta-analysis [Title/Abstract] OR meta-analysis [Title/Abstract] OR meta-analyses [Title/Abstract] )

#4 ((#1) AND #2) AND #3
