# Supplementary material for: Acupuncture for cancer pain: a scoping review of systematic reviews and meta-analyses
Source: Front Oncol. 2023 May 15;13:1169458. doi: 10.3389/fonc.2023.1169458 (PMC10226720; doi:10.3389/fonc.2023.1169458)
Supplement: Supplementary file 3 [file DataSheet_3.docx]

Supplementary material 3 Characteristics of included reviews (n=25)

| First author | year | SR or/and MA | Age | Special population | intervention | Aims | RCTs only | Number of studies | Outcoms | Main results |
| --- | --- | --- | --- | --- | --- | --- | --- | --- | --- | --- |
| De-hui Li(19) | 2021 | MA | none | none | Acupuncture + medicine | evaluation the efficacy 、 evaluation the safety | Yes | 19 | pain relief rate, quality of life,adverse events,burst pain rate,Analgesic efficacy, duration of response | pain relief rate |
| Bei Dong(20) | 2021 | MA | adult | none | wrist-ankle acupuncture 、wrist-ankle acupuncture + analgesics | evaluation the efficacy | Yes | 13 | pain relief rate, pain score,adverse events | pain relief rate, pain score |
| Yihan He(21) | 2019 | MA | none | none | acupuncture 、acupressure | summary of existing evidence | Yes | 17 | the Brief Pain Inventory, Numerical Rating Scale, visual analogue scales (VAS), Verbal Rating Scale, and other validated instruments for assessing the intensity of pain | Pain intensity |
| Juan Yang(22) | 2020 | MA | adult | none | acupuncture | analyse currently available publications | No | 5 | pain relief rate | pain relief rate |
| Caiqiong Hu(23) | 2016 | MA | adult | none | acupuncture | evaluation the efficacy 、evaluation the safety | Yes | 20 | Analgesic efficacy,quality of life, patient satisfaction,hospital attendance, adverse events | Analgesic efficacy |
| Yulan Yang(24) | 2020 | MA | adult | none | auricular acupuncture、auricular acupuncture therapy+medicine | evaluation the efficacy 、evaluation the safety | No | 9 | Pain Score，quality of life，adverse events | pain relief rate |
| Carole A Paley(26) | 2015 | MA | adult | none | acupuncture | evaluation the efficacy | Yes | 5 | visual analogue scales (VAS)，patient satisfaction，quality of life，Analgesic efficacy，hospital attendance，adverse events | visual analogue scales (VAS) |
| Adam Hurlow(27) | 2012 | MA | adult | none | Transcutaneous electric nerve stimulation (TENS) | evaluation the efficacy | Yes | 3 | patient satisfaction，function，range of movement，quality of life，mood，pain coping，sleep，Analgesic efficacy，hospital attendance，adverse events | patient reported pain |
| Tae-Young Choi(28) | 2012 | MA | none | none | acupuncture、acupuncture+medicien | evaluation the efficacy | No | 15 | pain relief rate | pain relief rate |
| Carole A Paley(29) | 2012 | MA | adult | none | acupuncture、 Electroacupuncture、Auricular acupuncture、 | evaluation the efficacy | Yes | 3 | visual analogue scales (VAS), numerical rating scales，pain relief rate，patient satisfaction，quality of life， Analgesic efficacy，hospital attendance，adverse events | visual analogue scales (VAS) |
| Carole A Paley(30) | 2011 | MA | adult | none | acupuncture | evaluation the efficacy | Yes | 3 | visual analogue,numerical rating scales,visual analogue scales (VAS), patient satisfaction, quality of life, Analgesic efficacy, hospital attendance,adverse events | visual analogue scales (VAS) |
| Karen A Robb(31) | 2008 | MA | adult | none | Transcutaneous electric nerve stimulation (TENS) | evaluation the efficacy | Yes | 2 | visual analogue scales (VAS), numerical rating scales,patient satisfaction,function,range of movement,quality of life,mood,pain coping,sleep,Analgesic efficacy,hospital attendance ,adverse events | visual analogue scales (VAS) |
| Hyangsook Lee(32) | 2005 | MA | none | none | acupuncture、auricular acupuncture、electroacupuncture | summary of existing evidence | Yes | 7 | visual analogue scales (VAS),patient's verbal assessment,plasma leucineenkephalin level | visual analogue scales (VAS) |
| Pu Yang(34) | 2021 | MA | adult | none | acupuncture+ three-step analgesia medicine | evaluation the efficacy 、evaluation the safety | Yes | 16 | pain relief rate,Time to effect, Analgesic efficacy, adverse events | pain relief rate |
| Hao Peng(35) | 2010 | MA | none | none | acupuncture、acupuncture + medicine | evaluation the efficacy | Yes | 7 | pain relief rate,Visual analogue scale,digital pain score grade, McGill pain score | pain relief rate |
| Jianfeng Wang(36) | 2020 | MA | adult | none | acupuncture+three-step analgesia medicine、 fire needle+ three-step analgesia medicine、electroacupuncture+ three-step analgesia medicine | evaluation the efficacy 、evaluation the safety | Yes | 14 | pain relief rate, degree of pain after treatment, quality of life, adverse events | pain relief rate |
| Jie Zhou(37) | 2014 | MA | none | none | Acupoint injection、Ashi point injection、ear point injection、acupoint injection+other drugs | evaluation the efficacy 、evaluation the safety | Yes | 13 | pain relief rate, drug onset time, maintenance time, adverse events | pain relief rate |
| Zou Yu(38) | 2018 | MA | none | none | acupoint application +analgesics | evaluation the efficacy 、evaluation the safety | No | 17 | Analgesic efficacy, adverse events, quality of life, morphine rescue rate, morphine consumption, sulfuric acid, morphine sustained-release tablet dosage | curative effect |
| Sun Ge(39) | 2011 | MA | none | none | wrist-ankle acupuncture +medicine | evaluation the efficacy | No | 21 | pain relief rate | Symptom improvement rate |
| Zheng Yi(40) | 2014 | MA | none | none | wrist-ankle acupuncture 、wrist-ankle acupuncture +medicine | evaluation the efficacy | No | 5 | Symptom scores, adverse events | Symptom improvement rate |
| Zhou Jie(41) | 2014 | MA | none | none | Auricular acupuncture+medicine、auricular acupuncture pills+medicine、auricular injection+medicine | evaluation the efficacy 、evaluation the safety | Yes | 8 | Pain score, adverse events | pain relief rate |
| HU Cai-qiong(42) | 2016 | MA | none | none | acupuncture、Acupuncture+medicine | evaluation the efficacy 、evaluation the safety 、summary of existing evidence | Yes | 20 | Analgesic efficacy, quality of life, patient satisfaction, hospital attendance, adverse events | Analgesic efficacy |
| SUN Qi-zhe(43) | 2013 | MA | none | none | Acupuncture + acupoint injection | evaluation the efficacy | Yes | 4 | valid, invalid | curative effect |
| BIAN Shuang-lin(44) | 2020 | MA | none | none | Acupuncture+three-step analgesia medicine | evaluation the efficacy 、evaluation the safety | No | 9 | Analgesic efficacy, pain intensity score, adverse events | Analgesic efficacy |
| CHEN Ting-yu(45) | 2019 | MA | none | none | Ashi points application+medicine | evaluation the efficacy | Yes | 16 | pain relief rate,symptom control, visual analog scale (VAS), adverse events, Karnofsky score (KPS). | pain relief rate |
